# Predisposing Factors and Outcomes After Prolonged Admission Following Hip Fracture

**DOI:** 10.7759/cureus.6044

**Published:** 2019-10-31

**Authors:** Kunal Mohan, Prasad Ellanti, Omar Hadidi, David C Moore, Niall Hogan, Tom McCarthy

**Affiliations:** 1 Orthopaedics, St. James's Hospital, Dublin, IRL; 2 Orthopaedics and Trauma, St. James's Hospital, Dublin, IRL

**Keywords:** hip, fracture, admission, prolonged, length of stay (los)

## Abstract

Introduction

Hip fractures are increasingly prevalent and can result in substantial morbidity, mortality, and cost. Despite the existence of enhanced management strategies, prolonged hip fracture admissions persist. This study’s objective was to ascertain characteristics associated with a prolonged length of stay (LOS) and quantify return to baseline once discharged.

Methods

A retrospective audit of hip fractures over a four-year period was conducted, identifying patients with a LOS over 100 days. Demographics, comorbidities, pre- and post-admission function, and status were assessed. Patients sustaining inpatient hip fractures were excluded to negate the effect of initial admission on LOS.

Results

Seven hundred and eleven hip fractures were treated, of which 48 (6.8%) were suitable for inclusion. The patients' median age and LOS was 83.5 years and 153 days, respectively. Preoperative American Society of Anesthesiologists - Physical Status (ASA-PS) Grades II and III predominated at 41.7% and 39.6%, respectively. Eighteen of patients had a diagnosis of dementia before admission, increasing to 29 on discharge (P = 0.0026). One patient was in long-term care prior to admission, rising to 30 on discharge (P < 0.0001), with only 25.6% returning to pre-admission residential status (P < 0.0001). Nineteen patients were mobilising unaided prior to admission, decreasing to only two following discharge, with a mere 37.1% returning to their pre-admission mobility baseline (P < 0.0001).

Discussion

Hip fracture patients with multiple comorbidities or a diagnosis of dementia were most likely to have a prolonged LOS which, in turn, impacted upon return to baseline mobility, cognitive status, and independence. Early identification and management of this cohort may help reduce the potential disease burden and economic effects that a prolonged LOS creates.

## Introduction

Hip fractures are common and account for up to 38% of all fractures encountered [1]. As a result, hip fractures can create a heavy cost burden, with the United Kingdom (UK) costs of hip fracture management significant at an estimated 1.1 billion pounds per annum [2].

The incidence of hip fractures is further expected to rise in the coming years as a result of an increasingly elderly population, with an overall global increase in hip fractures from 1.66 million in 1990 to 6.26 million by 2050 predicted [3-5]. Hip fractures are a known source of significant morbidity and mortality amongst the elderly population, particularly in those suffering from multiple comorbidities or limited ambulatory status. As such, this projected increase may create a growing burden on both patients and healthcare resources alike [5-6].

The predicted increase in hip fracture prevalence, in addition to the complex nature of hip fracture patients, has necessitated the requirement for streamlined management strategies in order to optimise patient outcomes and reduce potential fracture burden. These ideally consist of a multidisciplinary approach to initial stabilisation and patient optimisation, prompt surgical management when appropriate, and early patient mobilisation with the objective of facilitating return to the pre-fracture residence and activity [7]. The emergence of targeted national guidelines for comprehensive, multidisciplinary hip fracture management appears to have positively impacted patient outcomes, as well as leading to an overall improvement in the efficacy of hip fracture care [8-9].

This has been reflected in a number of parameters, one of which is the overall length of inpatient stay following hip fracture. The current mean length of inpatient stay following hip fracture ranges between 20 and 30.8 days, with a statistically significant reduction of length of stay (LOS) having been found following the implementation of streamlined hip fracture guidelines [9-11]. Prolonged inpatient hospital length of stay has been associated with poorer outcomes, particularly in elderly patient groups, with an overall functional decline and negative impact on quality of life associated with a protracted length of stay in this patient cohort; hence, amelioration of this parameter is of particular interest in the typical elderly hip fracture population [12]. The importance of this is further strengthened by the established cost-preserving benefits associated with decreasing length of hospital stay, particularly in the current climate of limited healthcare resources [13].

While the majority of hip fracture patients are typically discharged in a timely manner, with 84% and 98% of patients discharged within 30 and 90 days of admission, respectively, a small cohort of protracted inpatient stays following hip fracture continue to persist [10]. These patients are anecdotally associated with inferior outcomes and, in the context of the substantial costs associated with each hospital bed day, can create a severe burden on hospital resources [10, 14].

Thus, the objective of this study was to attempt to identify the characteristics potentially predisposing patients to a prolonged length of hospital stay following hip fracture, as well as to quantify the overall impact on mobility and return to baseline following a protracted admission in this rare but vulnerable patient group.

## Materials and methods

A retrospective, observational, single-centre audit of patients presenting with an acute hip fracture was undertaken, identifying those presenting over a four-year period, selected to ensure an appropriate representation of the typical patient group. All patients with an overall inpatient length of stay following hip fracture over 100 days were identified through a prospectively maintained hospital inpatient database and subsequently underwent clinical note and electronic hospital record review. Patients who sustained an inpatient hip fracture were excluded in order to negate the potential effect that the initial reason for admission may have on the overall LOS and post-discharge outcomes. An inpatient hospital length of stay of 100 days has previously been described in clinical healthcare literature as being sufficiently reflective of an abnormally prolonged length of stay. As such, it was selected as the minimum length of stay required for patient inclusion in this hip-fracture-specific study with the idea that selecting a value significantly above the typical median length of stay following hip fracture (20 - 30.8 days) would allow for adequate identification of this study’s primary outcomes, namely, identifying the predisposing characteristics and the return to function in this patient group [10, 15].

Overall patient demographics for each patient suitable for inclusion, including duration of stay, age, and gender, were recorded and each sustained fracture was classified by both its anatomical location and side. Concomitant injuries sustained at the time of hip fracture were also noted. The comorbid status of each patient was quantified through detailing the nature of each underlying comorbidity. Additionally, the preoperative American Society of Anaesthesiologists Physical Status (ASA-PS) Scale, a marker of a patient's preoperative health status, was obtained on review of either the perioperative anaesthetic notes or assessment of the patient's underlying comorbidities at the time of admission [16]. The intraoperative analysis was comprised of both the date of surgery and the type of anaesthetic administered. Lastly, the residential, cognitive, and mobility status were recorded for each patient at the time of admission and at discharge.

Each of the above parameters were recorded in an anonymised fashion into a singular electronic data spreadsheet, and the initial assessment of data involved the qualitative analysis of noticeable changes or trends that occurred during the course of each inpatient stay. The additional quantitative interpretation was performed using Stata, v14 software (StataCorp LLC, College Station, Texas, USA) for statistical analysis. McNemar’s test, a version of Chi-squared testing utilised for evaluating the homogeneity of dichotomous variables within paired subjects, was utilised to assess the alteration in both mobility status and discharge location following an inpatient stay, with a p-value less than 0.05 considered statistically significant [17].

## Results

Seven hundred and eleven patients with hip fractures were seen in the hospital over a 48-month period, of which 56 (7.9%) had an overall inpatient episode stay of over 100 days. Eight of these patients sustained inpatient hip fractures and were subsequently excluded. Forty-eight patients (6.8%) admitted to hospital with a hip fracture with an associated length of stay over 100 days were thus suitable for inclusion, with a median age and length of stay of 83.5 years and 153 days, respectively (Table 1).

**Table 1:** Included Patient Demographics

Fracture Type	Female	Male	Total
Number of Patients (Percentage)	32 (66.7%)	16 (33.7%)	48
Median Age in Years (Range)	85.18 (55 - 95)	82.33 (71 - 96)	83.5 (55 - 96)
Median Length of Stay in Days (Range)	148 (103 - 288)	148.5 (105 - 367)	153 (103 - 367)

Hip fractures were more frequent on the left side in this patient group, occurring in 25 (52.1%) of patients. The pattern of hip fracture was recorded in 46 patients (95.8%), with the most common hip fracture pattern encountered in this group being an intertrochanteric hip fracture (occurring in 23 (47.9%) of patients) with the neck of the femur, subtrochanteric, and periprosthetic fractures occurring in 15 (32.6%), five (10.9%), and three (6.5%) patients, respectively. Six patients (12.5%) presented with other concomitant fractures, and 44 patients (91.7%) underwent surgical intervention for their fracture, with the type of surgical therapy selected dictated by the fracture pattern as deemed suitable by the primary operating surgeon. The type of anaesthetic and the postoperative location were identifiable in 28 patients (63.6%), with 24 patients (85.7%) operated upon under spinal anaesthetic and only one patient (2.3%) requiring transfer to a higher dependency bed postoperatively.

**Figure 1:**
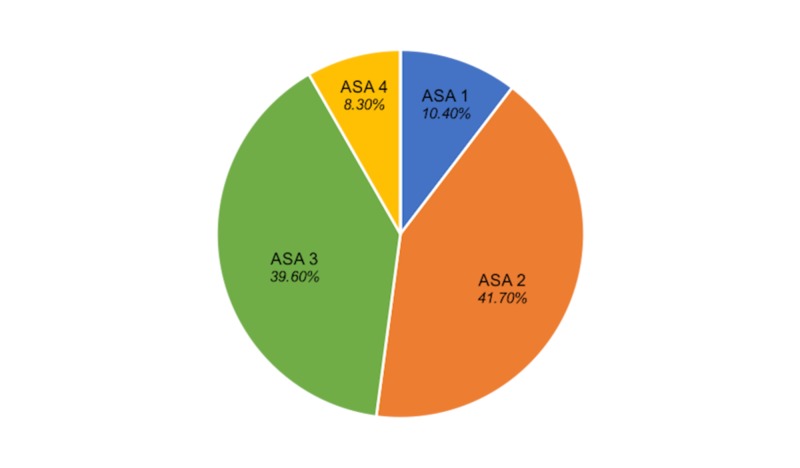
Included Patient American Society of Anaesthetists (ASA) Grading

Overall ASA grading for this patient group at the time of surgery showed a predominance of mild and severe underlying systemic disease (Figure 1).

The median number of patient comorbidities on presentation was five, ranging from 0 to 15. The individual patient analysis found a high incidence of cardiac disease in this long-stay inpatient cohort, with a documented history of atrial fibrillation, ischaemic heart disease, and hypertension found in 16 (33.3%), 10 (20.8%), and 25 (52.1%) patients, respectively. Twenty-two patients (45.8%) were taking antiplatelet agents and eight (16.7%) were on anticoagulants. A diagnosis of dementia at the time of the presentation was also prominent, affecting 18 patients prior to hospital admission, with this significantly increasing to 29 patients at the time of discharge (P = 0.0026) (Table 2). 

**Table 2:** Results N/A: not available

Results	Pre-admission	Post-discharge	P-value
% Dementia	37.5%	60.4%	P = 0.0026
% Discharged to Long-term Care	2.6%	76.9%	P < 0.0001
% Returning to Pre-admission Residential Status	N/A	25.6%	P < 0.0001
% Returning to Pre-admission Mobility Status	N/A	37.1%	P < 0.0001

**Figure 2:**
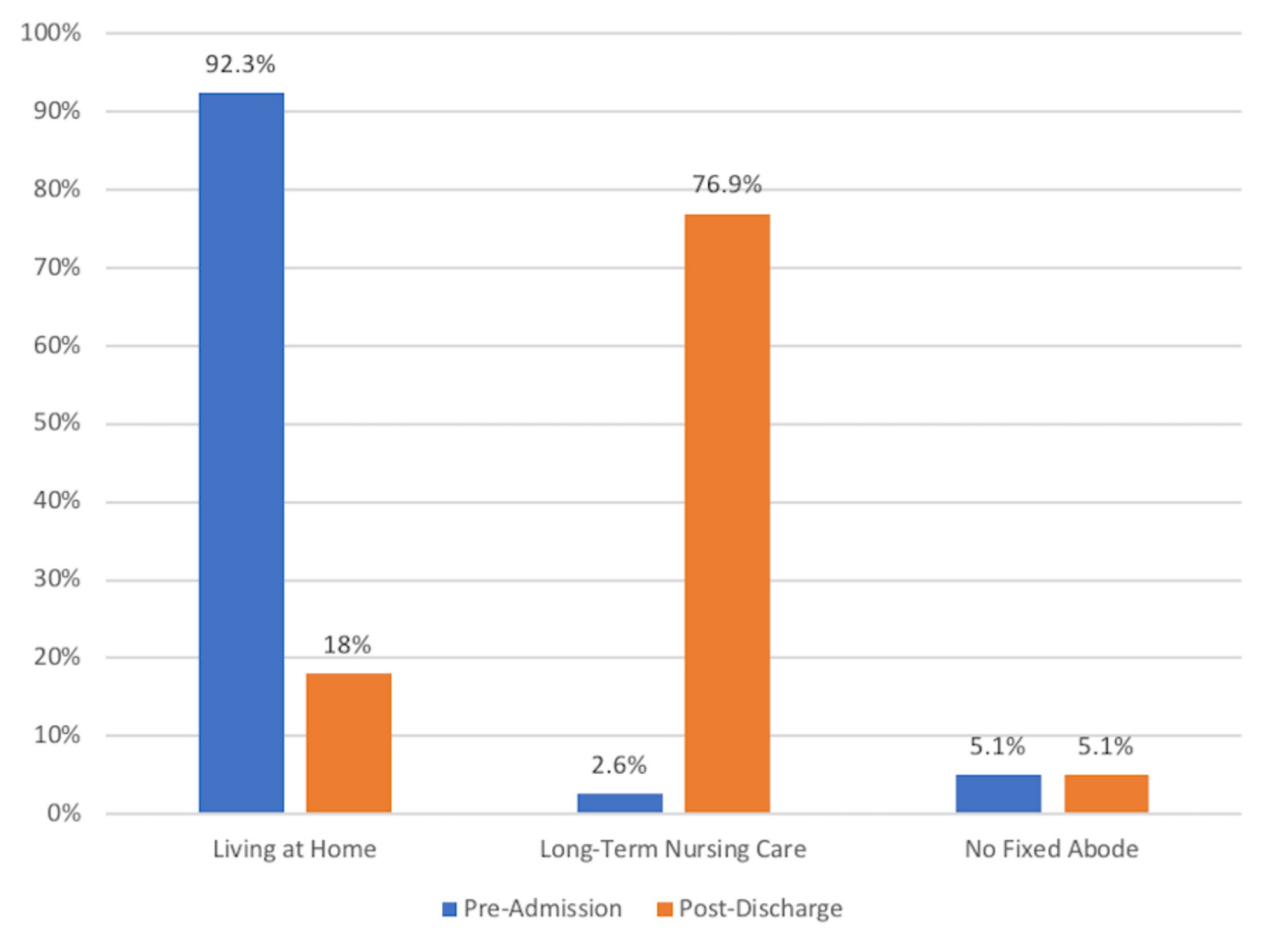
Patient Residential Status

Preadmission living status and eventual discharge location were documented in 39 patients (81.3%) and is further described in Figure 2. 

Thirty-six of these patients were living at home prior to admission, with 21 (58.3%) living alone. However, following admission, 29 of these patients were discharged to long-term care, with 16 (55.2%) having previously lived alone. Merely 10 patients returned to their pre-admission residential status (P < 0.0001) (Table 2).

**Figure 3:**
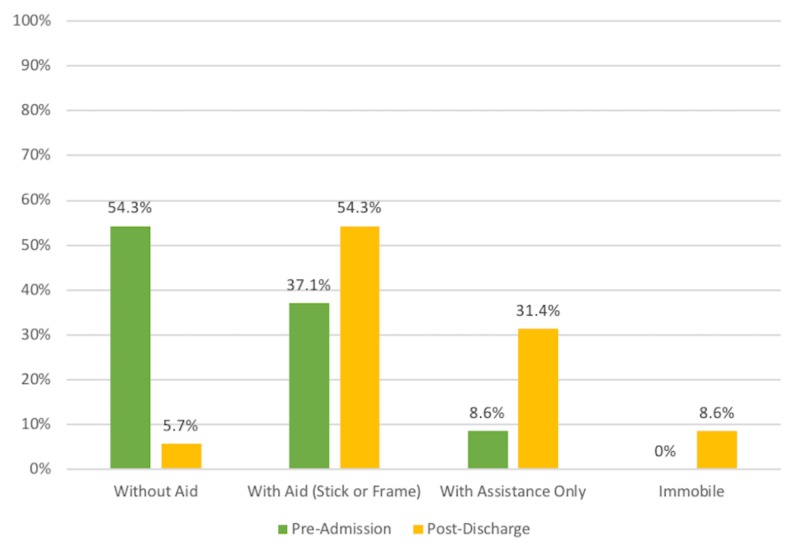
Patient Mobility Status

Mobility status was documented on both admission and discharge in 35 patients (72.9%) and is further illustrated in Figure 3.

Prior to admission, 19 patients were mobilising independently without aid, reduced substantially to only two patients on discharge. Overall, only 13 patients returned to their baseline mobility status following discharge (P < 0.0001) (Table 2).

## Discussion

In today’s aging population, hip fractures are increasing in frequency [3, 5]. Given that these fractures are a known source of morbidity and mortality in an elderly population, a particular focus has been made to create streamlined national guidelines in an attempt to optimise postoperative function and outcomes [5, 8]. Prolonged inpatient hospital stay has been shown to be particularly harmful in an elderly patient cohort, such as those typically encountered following hip fracture, and, as such, has been used as a representative marker of the efficacy of current hip fracture care [10, 12]. Current practice has ensured that a majority of patients are discharged promptly from acute hospitals, with 84% of patients discharged under 30 days, and the mean length of acute hospital stay reported at 20 and 21.6 days in the United Kingdom and the Republic of Ireland, respectively [10, 18]. However, a small number of hip fractures with a prolonged length of hospital stay continue to persist, and these patients anecdotally fare poorly. While predictive factors for a prolonged length of stay across all patients have previously been assessed, this vulnerable hip fracture cohort has not been specifically evaluated [19]. As such, the objectives of our study were two-fold: namely, to identify any potential predisposing factors for acute admission following hip fracture, as well as to quantify the functional outcomes in this prolonged stay hip fracture group.

Traditionally, elderly patients presenting to the hospital following a hip fracture typically suffer from multiple underlying comorbidities which, in turn, adds complexity to their treatment [20]. This usually is reflected in the ASA-PS score given to this group preoperatively, with Grades 2 and 3 reflective of mild or severe systemic disease, respectively, appearing to predominate (allocated to 92% of hip fracture patients) [10]. In our study of a protracted inpatient stay cohort, this appeared to also be the case, with 81.3% of patients allotted to these two categories. A higher ASA-PS score has previously been correlated with increased length of hospital stay, and while this pattern is well-represented in the long-stay cohort, it does not appear to be unique to this group alone [21].

However, a preoperative diagnosis that did appear to be more prevalent in the prolonged stay fracture cohort was dementia. While dementia is commonly seen in those sustaining a hip fracture (having been found to be present in up to 19.2% on admission), our study found that 37.5% of the prolonged stay cohort were diagnosed with dementia preoperatively [22]. Dementia has been associated with increased length of acute hospital stay previously, and the higher prevalence of this on admission in the prolonged stay cohort in comparison to all hip fractures seems to reflect this trend [22-24]. Furthermore, a prolonged length of stay following hip fracture appeared to also significantly increase the risk of developing dementia in those previously well otherwise, with 22.9% of prolonged stay patients newly diagnosed with the condition during their admission (P = 0.0026). This can lead to the inference that while dementia may be a potential risk factor for prolonged admission following hip fracture, the protracted stay superimposed upon the original trauma may additionally accelerate the diagnosis of dementia in a vulnerable patient group. 

Across all patients, hip fractures have been associated with potential limitations to postoperative mobility, with 50% to 70% of patients typically returning to their pre-fracture level of mobility following a hip fracture [25-26]. This, however, appeared to be more pronounced in the prolonged stay hip fracture cohort, with only 37.1% of patients returning to pre-admission mobility status (p < 0.0001). A deterioration in mobility status, particularly in the elderly patient, can lead to a substantial deterioration in health with an associated increase in healthcare resource utilisation [27]. As such, it appears that those with a prolonged length of stay following hip fracture may be at an increased risk of suffering the resultant ill effects of the loss of mobility. Given that the pre-admission mobility status in this prolonged stay hip fracture group appears to be favourable, with 54.3% of patients mobilising independently prior to fracture comparing favourably with the national rates of 48% and 37% in Ireland and the UK, respectively, for all fractures, one may deduce that the prolonged length of stay itself may be the primary factor for reduced post-discharge mobility [18].

Similar outcomes were detected in regards to changes in the residential status of patients. The vast majority of eventual long-stay patients assessed in this study originated from home prior to admission, again closely reflecting the general pattern seen across all hip fractures [10]. However, while between 60% and 84% of patients are typically expected to return home following hip fracture, only 19.4% of these long-stay patients returned home, with a statistically significant rise (p < 0.0001) in those being discharged to long-term nursing home care [28]. This would indicate that a prolonged length of stay following hip fracture may contribute to a substantial loss of independence and function in these patients, with a resultant impact on their overall health status [27]. A contributing factor to this may be the high preponderance (55.2%) of those living alone prior to admission in the group being discharged to long-term care. While demonstrating underlying independence, this may also be indicative of an at-risk group with limited underlying social supports, hence potentially increasing the need for higher postoperative levels of support.

Given the non-randomised descriptive and retrospective nature of our study, the significance of its findings may be limited. Furthermore, the lack of prolonged follow-up of the included patients may further potentiate its limitation, as it is feasible that more patients may have returned to baseline function or independence following hospital discharge. Nevertheless, we believe this study to be of importance as it allows for the identification of potential factors that, if acted upon early, may improve outcomes in this patient group, as well as illustrating the sequelae of prolonged hip fracture admission on patients following discharge. Awareness of the risk factors may allow for an early optimisation of patients with underlying dementia to help ameliorate outcomes. Similarly, being cognisant of the functional and societal limitations associated with prolonged length of stay may potentially drive further refinement and tailoring of preexisting hip fracture management pathways. In addition to the potential patient benefits of this, it may also reduce the potential economic burden associated with patients of impaired function and independence [27].

## Conclusions

In conclusion, hip fractures presenting with multiple comorbidities or a diagnosis of dementia seemed to be at higher risk of prolonged hospital length of stay. A prolonged length of stay following hip fracture appears to drastically impact subsequent cognitive status, return to baseline mobility, and residential status with the associated limitation in function and independence placing these vulnerable patients at an even higher risk of poorer outcomes. Early and appropriate identification and management of this cohort may help reduce the potential disease burden and associated economic effects a prolonged length of stay creates.
